# Diversity of cultivable protease-producing bacteria in sediments of Jiaozhou Bay, China

**DOI:** 10.3389/fmicb.2015.01021

**Published:** 2015-09-25

**Authors:** Xi-Ying Zhang, Xiao-Xu Han, Xiu-Lan Chen, Hong-Yue Dang, Bin-Bin Xie, Qi-Long Qin, Mei Shi, Bai-Cheng Zhou, Yu-Zhong Zhang

**Affiliations:** ^1^State Key Laboratory of Microbial Technology, Shandong UniversityJinan, China; ^2^Marine Biotechnology Research Center, Shandong UniversityJinan, China; ^3^State Key Laboratory of Marine Environmental Science, Xiamen UniversityXiamen, China

**Keywords:** protease-producing bacteria, diversity, Jiaozhou Bay, extracellular proteases, protease inhibitor tests

## Abstract

Although protease-producing bacteria are key players in the degradation of organic nitrogen and essential for the nitrogen recycling in marine sediments, diversity of both these bacteria and their extracellular proteases is still largely unknown. This study investigated the diversity of the cultivable protease-producing bacteria and their extracellular proteases in the sediments of the eutrophied Jiaozhou Bay, China through phylogenetic analysis and protease inhibitor tests. The abundance of the cultivable protease-producing bacteria was up to 10^4^ cells/g in all six sediment samples. The cultivated protease-producing bacteria mostly belonged to the phyla *Proteobacteria* and *Firmicutes* with the predominant genera being *Photobacterium* (39.4%), *Bacillus* (25.8%), and *Vibrio* (19.7%). Protease inhibitor tests revealed that extracellular proteases secreted by the bacteria were mainly serine proteases and/or metalloproteases with relatively low proportions of cysteine proteases. This study represents the first comprehensive analysis on the diversity of protease-producing bacteria and their extracellular proteases in sediments of a eutrophic bay.

## Introduction

Organic matters deposited at the sea floor, mostly in the polymeric and particulate forms, serve as the main nitrogen sources in sediments (Thamdrup and Dalsgaard, 2008). Microbial enzymatic hydrolysis of these nitrogenous macromolecules is essential for the mineralization of sedimentary organic nitrogen (SON) and the benthic nutrients recycling (Talbot and Bianchi, 1997; Fabiano and Danovaro, 1998; Herbert, 1999; Patel et al., 2001; Thamdrup and Dalsgaard, 2008; Arnosti, 2011; Arnosti et al., 2014). As proteins constitute large fractions of marine organic matters (Wakeham et al., 1997; Thamdrup and Dalsgaard, 2008; Lloyd et al., 2013; Moore et al., 2014), protease-producing bacteria are recognized as key players in the microbial degradation of SON (Herbert, 1999; Zhao et al., 2008, 2012; Chen et al., 2009; Zhou et al., 2009). They secrete extracellular proteases to hydrolyze complex proteinaceous substances into small peptides and amino acids suitable for cellular uptake (Zhao et al., 2008, 2012), initiating the mineralization of SON and driving the nitrogen cycle in marine ecosystem. Despite of their ecological and biogeochemical importance, there are only few studies to date investigating the diversity of sedimentary protease-producing bacteria and their extracellular proteases. Olivera et al. (2007) screened 19 protease-producing bacteria from sub-Antarctic sediments and found them be affiliated with the genera *Pseudoalteromonas, Shewanella, Colwellia, Planococcus*, and the family *Flavobacteriaceae*. We screened 78 and 105 protease-producing bacteria, respectively, from deep sea sediments of the South China Sea and coastal sediments of King George Island, Antarctica and analyzed the diversity of these bacteria and their extracellular proteases (Zhou et al., 2009, 2013). Results revealed that the cultivable protease-producing bacteria from deep sea sediments of the South China Sea were mainly affiliated with the class *Gammaproteobacteria*, while those from Antarctica coastal sediments mainly with the phyla *Actinobacteria, Firmicutes, Bacteroidetes*, and *Proteobacteria*, and extracellular proteases from these sedimentary protease-producing bacteria were almost all serine proteases or metalloproteases. Considering that marine environments are extremely diverse and complex, there may exist more abundant or complicated diversity of proteases-producing bacteria and their extracellular proteases in other marine regions, especially in tropical or temperate costal regions characterized by high organic matters input and more anthropogenic activities.

Jiaozhou Bay is located on the southern coast of Shandong Peninsula of China, the western coast of the Yellow Sea, and is a typical temperate semi-enclosed bay with a surface area of 362 km^2^ and an average water depth of 7 m (Li et al., 2008). The bay is connected to the Yellow Sea through a narrow channel (~2.5 km in width) at its entrance with average water residence time being 52 days (Liu et al., 2004). In the past four decades, with rapid socio-economic developments in the surrounding areas, Jiaozhou Bay has been greatly influenced by human activities and excess nutrient discharges from surrounding rivers, sewage processing plants, mariculture fields, and industrial and agricultural activities made parts of the bay hypernutrified (Liu et al., 2005, 2010; Dai et al., 2007; Dang et al., 2009). Several studies have investigated the diversity and community structure variations in response to changes in environmental conditions of the total sediment bacteria or specific functional groups involved in the key steps of the nitrogen cycling in the eutrophied Jiaozhou Bay using molecular techniques (Dang et al., 2009, 2010a,b; Liu et al., 2015a). But till now, there have been no report to investigate the diversity of protease-producing bacteria and their extracellular proteases in sediments of this eutrophic bay. In this study, sediment samples were collected from six stations with distinct environmental characteristics in Jiaozhou Bay, from which protease-producing bacteria were further screened. Diversity of both the cultivable protease-producing bacteria and the proteases they produced was subsequently studied, by the 16S rRNA gene sequence analysis and the protease inhibitor tests, respectively.

## Materials and methods

### Sample collection and geochemical characteristics

**Figure 1:**
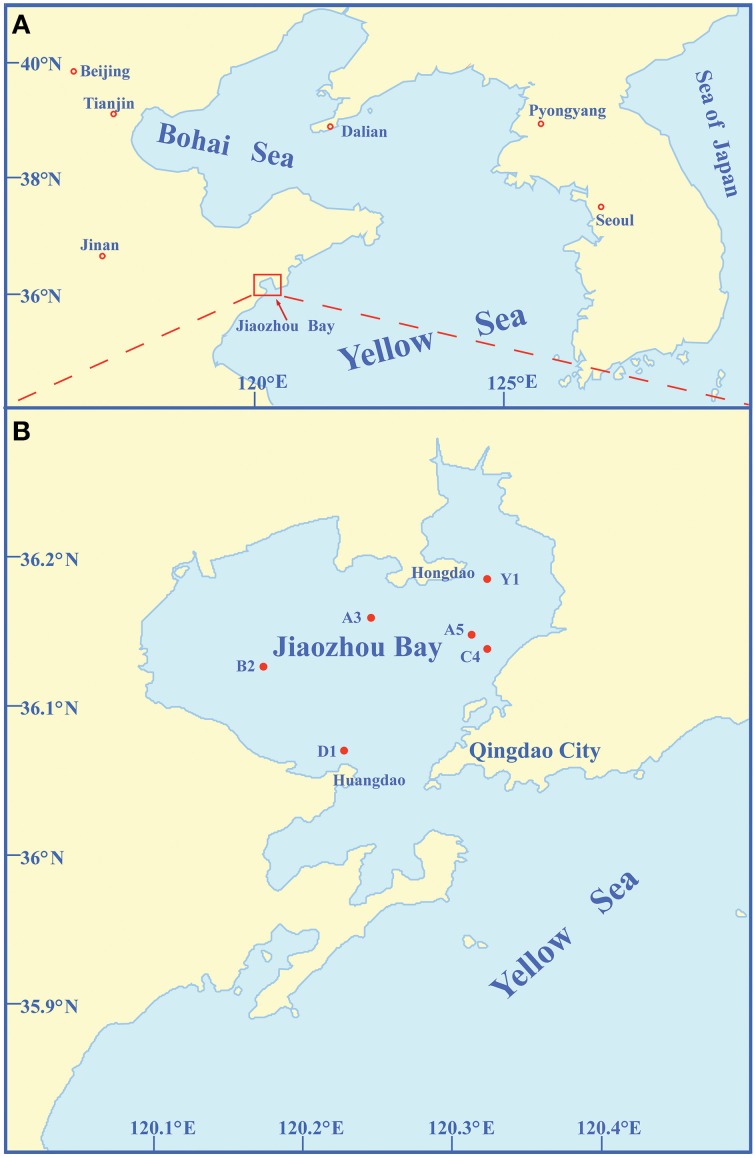
**Geographic location of Jiaozhou Bay (A) and sampling stations in Jiaozhou Bay (B)**.

Sediment samples were collected from six stations (A5, C4, Y1, A3, B2, and D1) of Jiaozhou Bay using a stainless steel 0.05-m^2^ Gray O'Hara box corer on September 2, 2008 (Figure 1, Table 1). These stations with different water depths (4.0–12.8 m) are located in different regions of Jiaozhou Bay (Figure 1, Table 1). Except for D1 station at the entrance of Jiaozhou Bay, other stations are in the inner bay: stations A5, C4, and Y1 are in the east coastal area of Jiaozhou Bay, the most hyper-nutrified and polluted part of the bay (Dang et al., 2009, 2010a); station A3 is in the mid-north of the bay; station B2 is in the mid-west and is the less-polluted station compared to the others (Dang et al., 2009; Liu et al., 2015a). Replicate surface sediment subcore samples (5 cm depth) were taken aseptically using sterile 60-ml syringes (luer end removed) and stored in airtight sterile plastic bags at 4°C for screening bacterial strains or at −80°C for environmental analysis. Temperatures and pH of the surface sediments were measured *in situ*. Organic carbon (OrgC) and nitrogen (OrgN) contents in the samples were analyzed using a PE 2400 Series II CHNS/O analyzer (Perkin Elmer, USA).

**Table 1:** **Characteristics of the sampling stations**.

**Station**	**Location (E, N)**	**Depth (m)**	**Temperature (°C)**	**pH**	**OrgC (%)**	**OrgN (%)**	**C/N**
A3	120.250°, 36.158°	5.7	24.8	8.05	1.88	0.04	47
A5	120.313°, 36.148°	5.9	24.7	8.11	0.77	0.11	7
B2	120.172°, 36.124°	4.0	24.3	8.03	1.23	0.15	8.2
C4	120.324°, 36.139°	12.8	24.6	8.05	1.39	0.02	69.5
D1	120.224°, 36.070°	11.4	23.8	8.04	0.78	0.06	13
Y1	120.322°, 36.183°	5.3	24.5	8.03	1.02	0.08	12.75

### Screening of protease-producing bacteria from sediment samples

Protease-producing bacteria were screened from the sediment samples using the dilution-plate method on a screening medium containing 0.2% yeast extract, 0.3% casein, 0.5% gelatin, 1.5% agar, and artificial sea water (pH 8.0), as described by Zhou et al. (2009). Artificial sea water (ASW) was prepared using the Sigma sea salt (3%). In brief, approximate 1 g (wet weight) sediment sample was 10-foldedly serially diluted to 10^−5^ dilution with ASW. Aliquots of 100 μl of the diluted samples (10^−1^–10^−5^ dilution) were spread on the screening medium and incubated at 15°C until colonies with clear hydrolysis zone were visible. Different-looking colonies with hydrolysis zone were selected and further purified by streaking on the same medium for several times. Strains purified were cultivated in a liquid medium containing 0.5% tryptone, 0.1% yeast extract, and artificial seawater (TYS broth) at 15°C and preserved at −80°C in TYS broth supplemented with 15% (v/v) glycerol.

### PCR amplification, sequencing of 16S rRNA genes and phylogenetic analysis

Genomic DNAs of isolates were extracted using a bacterial genomic DNA isolation kit (BioTeke, China). The 16S rRNA gene were PCR-amplified with the forward primer 27F (5′-AGAGTTTGATCCTGGCTCAG-3′) and the reverse primer 1492R (5′-GGTTACCTTGTTACGACTT-3′). The PCR products were ligated into pGEM-T vectors (Promega, USA) and further sequenced at Biosune Inc. (Shanghai, China). Two isolates were considered to be different strains if they possessed at least two nucleotide differences in their 16S rRNA gene sequences (Zhou et al., 2009). The sequence alignment and phylogenetic analysis were performed using MEGA version 5 (Tamura et al., 2011). Phylogenetic trees were constructed based on the Neighbor-Joining method (Saitou and Nei, 1987) and using the Kimura two-parameter model (Kimura, 1980).

### Hydrolysis ability of proteases to different substrates

Three kinds of solid media were prepared by adding 0.5% (*w*/*w*) casein, 0.5% (*w*/*w*) gelatin, or 0.5% (*w*/*w*) elastin powder into a basic medium containing 0.2% yeast extract, 1.5% agar and ASW. Strains with the ability to form clear hydrolysis zone on the screening medium were streaked on the three media and incubated at 15°C for 4 days. For each strain, the diameters of its colony and the hydrolytic zone it formed were measured and the ratio of the hydrolytic zone diameter to the colony diameter (hydrolytic zone/colony, H/C) was then calculated (Zhou et al., 2009).

### Effect of different inhibitors on protease activity

Protease-producing strains were cultivated in the liquid screening medium at 15°C, 200 r/m for 4 days. After bacterial cells were removed by centrifugation at 12,000 × g, 4°C, the protease activity of the resulting supernatant was determined as described before (Chen et al., 2003). One unit of enzyme activity was defined as the amount of enzyme that catalyzed the formation of 1 μg tyrosine per minute. The supernatant properly diluted with 50 mM Tris-HCl (pH 8.0) was incubated with 1.0 mM phenylmethylsulfonyl fluoride (PMSF, Sigma), 1.0 mM 1, 10-phenanthroline (OP, Sigma), 10 mM iodoacetic acid (Sigma), or 0.1 mM Pepstatin A (Merk) at 20°C for 20 min. After incubation, the residue protease activity of every sample was measured as previously described (Chen et al., 2003). The activity of the sample without any inhibitor (the control) was set as 100% activity, and the relative activity (%) of the samples was calculated. The inhibition ratio was taken as the result of the control activity minus the relative activity of a sample (Zhou et al., 2009).

### Nucleotide sequence accession numbers

The sequences of 16S rRNA gene sequences obtained in this study were deposited in GenBank under the accession numbers from JX134415 to JX134480.

## Results

### Sediment characteristics

All sediment samples exhibited slightly alkaline pH (8.0–8.2). The content of OrgC and OrgN in the sediments ranged from 0.77 to 1.88% (OrgC) and 0.02–0.15% (OrgN), respectively. The highest values were found in station A3 (OrgC) and B2 (OrgN) while the lowest ones in stations A5 (OrgC) and C4 (OrgN). But the highest C/N ratio (69.5) was found in the station C4 and the lowest (7.0) in station A5.

### Screening of extracellular protease-producing bacteria from sediments

There were a number of colonies appearing on the screening plates of the 10^−1^–10^−3^ diluted samples after cultivation at 15°C for 2–5 days. Plate counts showed that the richness of cultivated bacteria in all samples reached 10^4^ cells/g and approximate 60% colonies produced clear hydrolytic zone. There was no obvious difference in the richness of the cultivable protease-producing bacteria among the stations although the OrgC and OrgN contents and the C/N ratios in these stations were different. Sixty-nine colonies able to form hydrolytic zone on the screening plates were purified for the subsequent phylogenetic identification.

### Diversity of the cultivable protease-producing bacteria from the sediments

**Figure 2:**
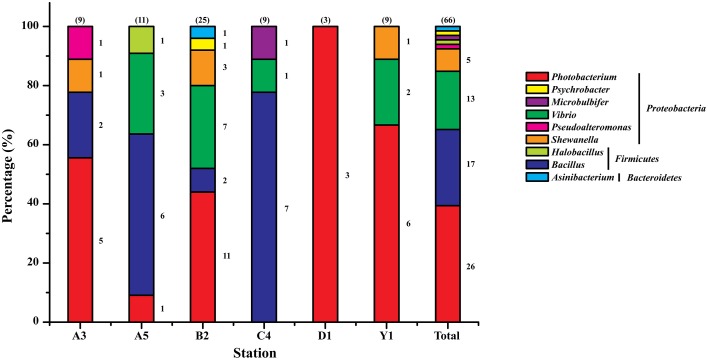
**Relative percentage abundance of the phylotypic groups of cultivable protease-producing bacteria isolated from six sampling stations in Jiaozhou Bay**. The total number of strains screened from each station is shown in the parenthesis above each column. Strain numbers per genus are shown at the right of sections of the columns.

The nearly full-length 16S rRNA genes of the 69 isolates were amplified and sequenced, based on which their phylogenetic affiliation was analyzed. Three isolates were considered to be the same strain because they had identical 16S rRNA gene sequences. Thus, a total of 66 protease-producing strains were obtained. With the exception of one strain belonging to the genus *Asinibacterium* in the phylum *Bacteroidetes*, all strains were affiliated with eight genera in the phyla *Proteobacteria* and *Firmicutes*, including *Photobacterium, Bacillus, Vibrio, Shewanella, Pseudoalteromonas, Halobacillus, Microbulbifer*, and *Psychrobacter*. Among them, *Photobacterium* (39.4%), *Bacillus* (25.8%), *Vibrio* (19.7%), and *Shewanella* (7.6%) were the major groups, while *Pseudoalteromonas, Halobacillus, Microbulbifer, Psychrobacter*, and *Asinibacterium*, all represented by only one strain, constituted a very minor fraction (< 8%) of the total bacteria. Meanwhile, *Photobacterium* was found to be present in 5 sediments and dominated in A3, B2, D1, and Y1 samples, while *Bacillus* presented in 4 sediments and dominated in C4 and A5 samples (Figure 2). *Photobacterium* and *Bacillus* represented the most frequently recovered and most abundant groups (43 of 66 strains) of the cultivated protease-producing bacteria in Jiaozhou Bay sediments. Furthermore, the protease-producing bacteria (belonging to six genera) cultivated from the B2 sample were apparently more diverse than those from other stations' samples. In contrast, protease-producing bacteria from the D1 sample were all affiliated with the genus *Photobacterium*, representing the least diverse community of the cultivated protease-producing bacteria among the six stations.

**Figure 3:**
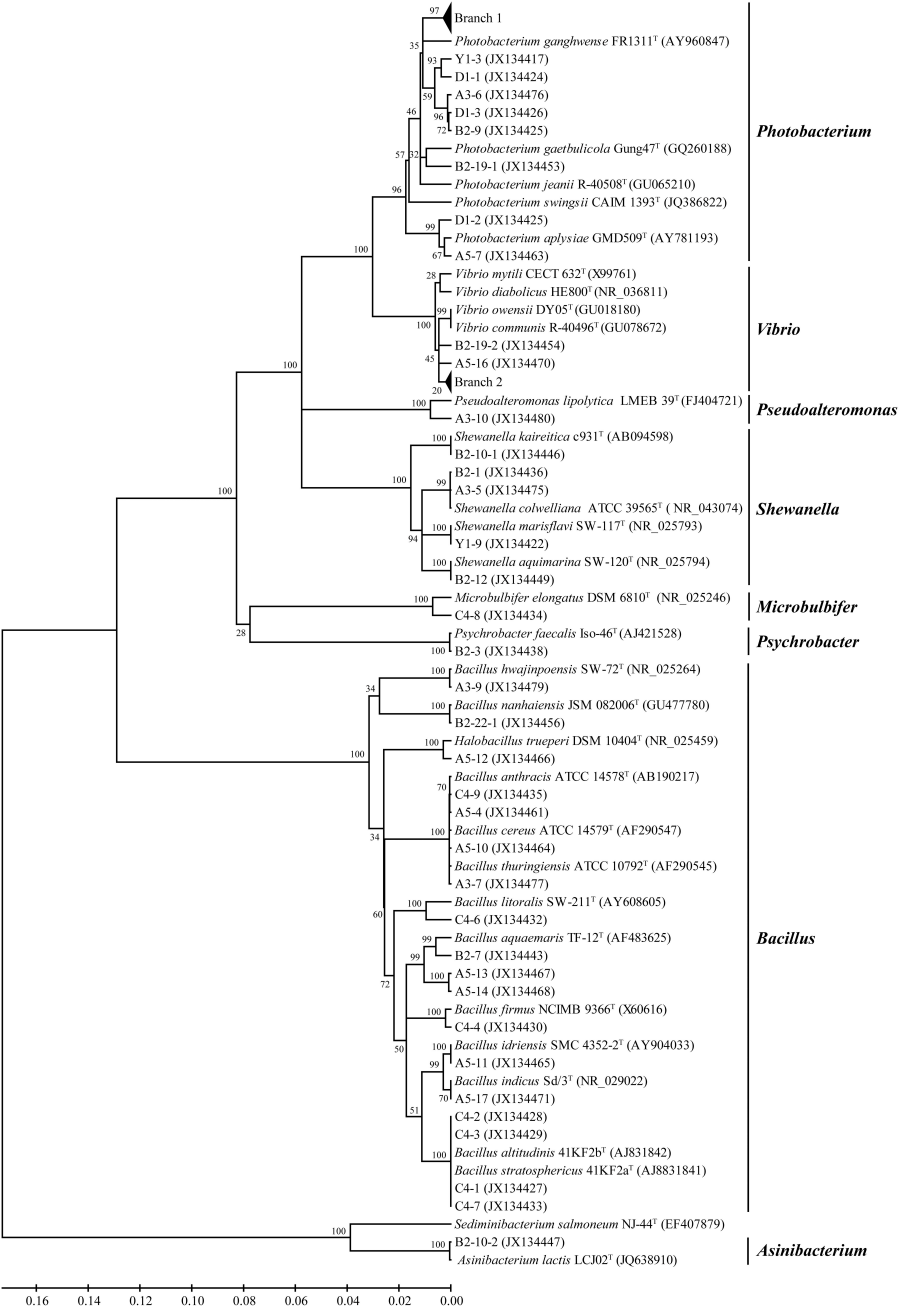
**Neighbor-joining tree of the protease-producing bacteria isolated from six stations in Jiaozhou Bay based on their 16S rRNA gene sequences**. Branch 1 indicates 18 *Photobacterium* strains closely related to *Photobacterium* sp. MA1-3 (JQ315889); Branch 2 indicates 11 *Vibrio* strains closely related to *Vibrio alginolyticus* (CP006718).

A neighbor-joining tree based on 16S rRNA gene sequences of the protease-producing strains was constructed to illustrate their phylogenetic relationship with different genera (Figure 3). Eighteen *Photobacterium* strains (recovered from 3 sediments) formed Branch 1 in Figure 3, all being closely related to *Photobacterium* sp. MA1-3 (JQ315889) isolated from an intertidal flat in Korea (Kim et al., 2012). Eleven *Vibrio* strains (recovered from 4 sediments) formed Branch 2 in Figure 3, all being closely related to *Vibrio alginolyticus* (CP006718), the dominant *Vibrio* species in seawater and farmed marine animals of the China coast (Liu et al., 2015b). In addition, strains D1-1 and D1-3 had distant relationship with all recognized *Photobacterium* species and may represent novel *Photobacterium* species, which merit further investigation.

### Diversity of the extracellular proteases from the screened bacteria

The diversity of the extracellular proteases of the screened protease-producing bacteria from Jiaozhou Bay sediments was investigated by analyzing the effects of different inhibitors on the protease activity (Table 2). PMSF (serine protease inhibitor), OP (metalloprotease inhibitor), iodoacetic acid (cysteine protease inhibitor), and Pepstatin A (aspartic protease inhibitor) were used to inhibit the activities of the proteases secreted by the screened strains to identify the types of these proteases. When cultivated in the liquid screening medium, of the 66 strains, only 28 strains affiliated with the genera *Photobacterium, Bacillus, Vibrio*, and *Shewanella* were able to produce enough extracellular proteases for activity inhibition analysis. Among the 28 strains, the protease activities of 16 strains were inhibited at the degree of 23–100% by PMSF, indicating that these strains all produced extracellular serine proteases at different levels; in particular, a high degree of inhibition (more than 90%) was observed in 6 strains, suggesting that these 6 strains mainly produce extracellular serine proteases. OP inhibited the protease activities of 18 strains by 11–86%, indicating that a majority of the screened strains produced extracellular metalloproteases. Iodoacetic acid inhibited the protease activities of 21 strains by 11–46%, indicating that some of the strains produce extracellular cysteine proteases in relatively low proportions. Meanwhile, the protease activities of a majority of the strains (17 of 28 strains) were inhibited by both OP and iodoacetic acid, indicating that these strains have the capacity to simultaneously produce metalloproteases and cysteine proteases. Several strains, including Y1-3, A5-16, B2-15-2, and B2-19-2, were even able to simultaneously produce serine proteases, metalloproteases, and cysteine proteases, because PMSF, OP, and iodoacetic acid all could inhibit the protease activities of these strains at significant levels. Moreover, Pepstatin A only had less than 12% or no inhibitory effect on the protease activities of all the tested strains, demonstrating that all these strains scarcely produced extracellular aspartic proteases.

**Table 2:** **Effects of inhibitors on the extracellular proteases secreted by strains from Jiaozhou Bay sediments**.

**Genera**	**Strains**	**Inhibition Ratio (%)^a^**			
		**PMSF (1 mM)**	**OP (1 mM)**	**P-A (0.1 mM)**	**Iodoacetic acid (10 mM)**
*Photobacterium*	A3-2	0.96	62.35	0	13.55
	A3-4	0	73.20	0	13.04
	A3-8	3.54	57.96	0.22	14.49
	A5-7	85.27	0	0	13.09
	B2-14	3.57	61.04	0	11.41
	B2-19-1	87.51	0	8.70	19.58
	B2-24	0	63.20	1.94	19.41
	B2-27	0	64.56	3.67	14.50
	D1-2	0	61.44	1.71	21.06
	Y1-1	0	65.08	1.19	17.13
	Y1-3	100	24.96	12.42	20.44
*Bacillus*	A3-7	1.62	69.95	0	13.39
	A3-9	25.84	79.80	8.83	0
	A5-13	0	76.40	0	21.35
	A5-14	28.25	80.68	8.26	0
	B2-7	84.19	5.32	0	0
	B2-22-1	87.10	2.43	0	0
	C4-2	95.11	0	0	19.23
	C4-7	0.62	67.09	0.59	35.67
	C4-9	27.89	85.66	0	0.21
*Vibrio*	A5-16	100	11.4	0	34.75
	B2-8	89.30	5.04	0.7	23.46
	B2-15-2	95.21	7.35	2.24	23.45
	B2-19-2	89.99	8.08	6.16	8.93
	C4-5	1.97	84.64	5.71	13.01
	Y1-5	100	2.22	5.41	24.47
	Y1-10	91.73	1.62	1.00	12.87
*Shewanella*	Y1-9	51.60	11.65	3.81	0

a The activity of a sample without any inhibitor was taken as control (100%). The inhibition ratio was taken as the result of control activity minus the relative activity of a sample with an inhibitor. PMSF, Phenylmethylsulfonyl fluoride; OP, 1,10-Phenanthroline; P-A, Pepstatin A.

In addition, the diversity of bacterial proteases from Jiaozhou Bay sediments were also investigated by analyzing the hydrolysis ability of the proteases to different proteins through measuring the H/C ratios of colonies on the plates containing gelatin, casein or elastin. Overall, judged from the abilities to form the hydrolytic zones on plates containing different proteinaceous substrates (Table 3), extracellular proteases from 56 strains (84.8% of the total strains) could hydrolyze casein and those from 56 strains could hydrolyze gelatin; in contrast, extracellular proteases from only 17 strains (25.8% of the total strains) were able to hydrolyze elastin. Moreover, the proteases from different strains had very different hydrolytic abilities to casein, gelatin and elastin, because they had apparently different H/C ratios on the plates containing the above three substrates (Table 3). Particularly, the extracellular proteases from strains A3-1, B2-26, Y1-3, Y1-6, and Y1-8 of *Photobacterium* and A3-9 of *Bacillus* showed high caseinolytic activity with the H/C ratios more than 6, while those from strains B2-4, B2-14, B2-17, B2-24, B2-26, B2-27, Y1-3, Y1-6, Y1-7, and Y1-8 of *Photobacterium*, C4-2 and C4-9 of *Bacillus*, A5-12 of *Halobacillus* and A3-10 of *Pseudoalteromonas* had high gelatinolytic activity with the H/C ratios more than 10. However, the extracellular proteases from most of the strains had no or very low elastinolytic activity with the H/C ratios less than 3 except those from strains A3-8, B2-4, B2-24, and B2-7, which had moderate elastinolytic activity with the H/C ratios between 3 and 5. In addition, the extracellular proteases from some strains, such as B2-24, B2-7, and Y1-5, had hydrolytic activity to all the three substrates (Table 3). In all, difference in the hydrolysis ability to the three proteins of the extracellular proteases from the screened strains, inferred from the variation of the H/C ratios, reflected their difference in kind or in specificity toward the three proteins.

**Table 3:** **The H/C ratios of the strains on the plates containing casein, gelatin, or elastin**.

**Genera**	**Strains**	**H/C ratio^a^**			**Genera**	**Strains**	**H/C ratio**		
		**Casein**	**Gelatin**	**Elastin**			**Casein**	**Gelatin**	**Elastin**
*Photobacterium*	A3-1	6.09	12.3	0	*Bacillus*	A3-7	1.83	0	0
	A3-2	3.64	1.44	Thin		A3-9	6.12	4.93	0
	A3-4	Thin^b^	7	Thin		A5-4	1.83	0	0
	A3-6	5.97	0	0		A5-10	Thin	Thin	0
	A3-8	1.65	Thin	3.1		A5-11	0	4.25	0
	A5-7	4.38	5.74	0		A5-13	Thin	0	0
	B2-2	3.29	6	0		A5-14	2.77	Thin	Thin
	B2-4	5.7	11.18	4.34		A5-17	Thin	Thin	0
	B2-9	Thin	Thin	2.53		B2-7	4.09	7.08	4.9
	B2-11	Thin	7.9	0		B2-22-1	4.0	0	1.79
	B2-14	3.71	10.67	0		C4-1	Thin	0	0
	B2-17	0	23	0		C4-2	Thin	13.88	0
	B2-19-1	3.76	6.5	0		C4-3	0	Thin	0
	B2-20	0	Thin	0		C4-4	0	5.5	0
	B2-24	3.5	16.33	3.68		C4-6	0	5	0
	B2-26	7.17	10.09	0		C4-7	2.4	0	0
	B2-27	4.41	24.8	0		C4-9	2.2	10.18	Thin
	D1-1	Thin	0	0	*Vibrio*	A5-5	1.94	Thin	0
	D1-2	5.41	8.79	0		A5-15	Thin	3.43	0
	D1-3	Thin	Thin	Thin		A5-16	3.75	4.63	0
	Y1-1	3.65	4.78	0		B2-5-1	1.87	7.87	Thin
	Y1-2	1.57	4.75	0		B2-5-2	1.55	2.69	0
	Y1-3	7.0	11	0		B2-6	1.82	2.48	0
	Y1-6	6.25	21.05	Thin		B2-8	3	6.22	0
	Y1-7	0	11.25	Thin		B2-15-2	Thin	0	Thin
	Y1-8	6.18	22.10	0		B2-19-2	2.35	2.79	0
*Shewanella*	A3-5	2.68	2.26	0		B2-23	0	Thin	0
	B2-1	2.92	5.13	0		C4-5	3.5	Thin	0
	B2-10-1	2.8	3.01	0		Y1-5	1.47	2.31	1.63
	B2-12	2.65	Thin	0		Y1-10	1.45	2.61	0
	Y1-9	Thin	2.42	0	*Pseudoalteromonas*	A3-10	0	14.67	0
*Psychrobacter*	B2-3	5.33	0	0	*Halobacillus*	A5-12	Thin	12.67	Thin
*Asinibacterium*	B2-10-2	3.9	3.32	0	*Microbulbifer*	C4-8	0	2.23	0

a H/C ratio is the ratio of the hydrolytic zone diameter to the colony diameter of a colony on the plate.

b Thin represents a slight hydrolytic zone formed by the strain.

## Discussion

Protease-producing bacteria are indispensable participants in the processes of organic nitrogen decomposition and recycling in marine environments. However, information on their diversity and their extracellular proteases in most regions is lacking, particularly in China coast with intense nitrogen biogeochemical cycling (Dang et al., 2010a). In this study, the diversity and community composition of the cultivated protease-producing bacteria and the diversity of extracellular proteases secreted by these bacteria in sediments of Jiaozhou Bay, China were investigated, through culture-based analysis and protease inhibitor assays.

The culture-independent analysis (using the 16S rRNA gene clone library analysis) recently revealed that sediments of Jiaozhou Bay harbored extremely diverse bacteria belonging to 17 bacterial phyla, among which *Proteobacteria* (61.3% of the total sequences), especially *Gammaproteobacteria* (32.8%), constituted the most abundant group (Liu et al., 2015a). Correspondingly, in our study, 71.2% of the total cultivated protease-producing strains (47 of 66 strains) are *Gammaproteobacteria*. This is also in consistence with previous findings that *Gammaproteobacteria* dominated the cultivated protease-producing bacteria from the sediments of the South China Sea (Zhou et al., 2009) and from the sub-Antarctic sediments (Olivera et al., 2007). These findings suggest that *Gammaproteobacteria* may be important protease-producing bacteria widely distributed in various marine environments. Of the *Gammaproteobacteria* screened in this study, *Photobacterium* (26 strains, 55.3% of all *Gammaproteobacteria*) and *Vibrio* (13 strains, 27.7% of all *Gammaproteobacteria*) strains were found to be the most abundant and distributed in almost all the stations. *Photobacterium* and *Vibrio* are very close relatives affiliated within the *Vibrionaceae*. They are ubiquitous in marine environments and are often found to be associated with marine animals (Urbanczyk et al., 2011). Since Jiaozhou Bay is a traditional area for marine animal farming (like fish, shellfish and shrimp), the intense mariculture in this bay may partially account for the high occurrence of *Photobacterium* and *Vibrio* in the bay sediments. In addition, the richness (10^4^ cells/g) of the cultivated protease-producing bacteria in sediments of Jiaozhou Bay screened using selective plates in this study is much lower than those of the South China Sea sediments (10^6^ cells/g) and the sub-Antarctic sediments (10^5^ cells/g) (Zhou et al., 2009, 2013). The predominance of *Photobacterium* and *Vibrio* likely resulted in the relatively low richness of the cultivated protease-producing bacteria in sediments of Jiaozhou Bay, because the *Photobacterium*- and *Vibrio*-affiliated strains usually produce broad-range inhibitory compounds, which can inhibit the growth of other bacteria (Mansson et al., 2011).

Although *Firmicutes* was detected as a very minor phylum in Jiaozhou Bay sediments by the culture-independent method (Liu et al., 2015a), *Bacillus* was found to be one of the predominant genera in the screened protease-producing strains from Jiaozhou Bay sediments in this study, confirming that *Bacillus* strains are easily cultivated protease-producing bacteria. *Bacillus* spp. are usually a major fraction in the culturable heterotrophic bacterial communities of coastal areas owing to their strong adaptation abilities to the dynamic environmental conditions of these areas. As *Bacillus* strains are also widespread in terrestrial habitats and bays are the interfaces between marine and terrestrial environments, some protease-producing *Bacillus* strains in Jiaozhou Bay sediments are probably not indigenous to local marine environments but originate from surrounding terrestrial ones. Analogously, *Bacillus* also constituted one of the predominant groups of cultivated protease-producing bacteria from the sub-Antarctic coastal sediments (Zhou et al., 2013).

It is worth noting that the culturable protease-producing bacteria from station B2, the least polluted one among the six sampling stations in Jiaozhou Bay, were more diverse than those from other stations. However, in station A5, located in the most polluted area of the bay, the composition of the culturable protease-producing bacteria was quite different from that in station B2 although values of the content of OrgN in the two stations are close. Particularly, a much higher proportion of *Bacillus*, maybe of terrestrial or anthropogenic origin, could be observed in station A5. These suggest that pollutions from anthropogenic activities have significant impact on the community structure of the culturable protease-producing bacteria in Jiaozhou Bay sediments. As found in our previous studies (Zhou et al., 2009, 2013), serine- and metallo-proteases are the principal types of proteases secreted by bacteria from Jiaozhou Bay sediments. In addition, we found that, bacteria from Jiaozhou Bay sediments may secrete cysteine proteases in relatively low amounts according to the inhibitor tests. A variety of extracellular proteases can make bacteria efficiently hydrolyze diverse and complex proteinaceous substances in Jiaozhou Bay.

In summary, this study analyzed the diversity of cultivable protease-producing bacteria in the sediments of six typical stations in Jiaozhou Bay and the types of the extracellular proteases secreted by these bacteria. The results showed that *Photobacterium, Bacillus*, and *Vibrio* are the major cultivated protease-producing groups in Jiaozhou Bay sediments and serine- and metallo-proteases the principal extracellular proteases secreted by the bacteria. These findings shed light on the ecological functions of protease-producing bacteria and their extracellular proteases in coastal ecosystems, and are helpful in elucidating the degradation mechanism of SON. In addition, marine bacterial strains with potentially novel proteases are obtained. Our work on the bio-prospective from the screened strains for novel proteases is on the way.

### Conflict of interest statement

The authors declare that the research was conducted in the absence of any commercial or financial relationships that could be construed as a potential conflict of interest.

## References

[B1] Arnosti C. (2011). Microbial extracellular enzymes and the marine carbon cycle. Ann. Rev. Mar. Sci. 3, 401–425. 10.1146/annurev-marine-120709-142731 21329211

[B2] Arnosti C. Bell C. Moorhead D. L. Sinsabaugh R. L. Steen A. D. Stromberger M. (2014). Extracellular enzymes in terrestrial, freshwater, and marine environments: perspectives on system variability and common research needs. Biogeochemistry 117, 5–21. 10.1007/s10533-013-9906-5

[B3] Chen X. L. Xie B. B. Bian F. Zhao G. Y. Zhao H. L. He H. L.. (2009). Ecological function of myroilysin, a novel bacterial M12 metalloprotease with elastinolytic activity and a synergistic role in collagen hydrolysis, in biodegradation of deep-sea high-molecular-weight organic nitrogen. Appl. Environ. Microbiol. 75, 1838–1844. 10.1128/AEM.02285-08 19201976 PMC2663233

[B4] Chen X. L. Zhang Y. Z. Gao P. J. Luan X. W. (2003). Two different proteases produced by a deep-sea psychrotrophic bacterial strain, *Pseudoaltermonas* sp. SM9913. Mar. Biol. 143, 989–993. 10.1007/s00227-003-1128-2

[B5] Dai J. Song J. Li X. Yuan H. Li N. Zheng G. (2007). Environmental changes reflected by sedimentary geochemistry in recent hundred years of Jiaozhou Bay, North China. Environ. Pollut. 145, 656–667. 10.1016/j.envpol.2006.10.005 17140715

[B6] Dang H. Chen R. Wang L. Guo L. Chen P. Tang Z.. (2010a). Environmental factors shape sediment anammox bacterial communities in hypernutrified Jiaozhou Bay, China. Appl. Environ. Microbiol. 76, 7036–7047. 10.1128/AEM.01264-10 20833786 PMC2976235

[B7] Dang H. Li J. Chen R. Wang L. Guo L. Zhang Z.. (2010b). Diversity, abundance, and spatial distribution of sediment ammonia-oxidizing Betaproteobacteria in response to environmental gradients and coastal eutrophication in Jiaozhou Bay, China. Appl. Environ. Microbiol. 76, 4691–4702. 10.1128/AEM.02563-09 20511433 PMC2901714

[B8] Dang H. Wang C. Li J. Li T. Tian F. Jin W.. (2009). Diversity and distribution of sediment nirS-encoding bacterial assemblages in response to environmental gradients in the eutrophied Jiaozhou Bay, China. Microb. Ecol. 58, 161–169. 10.1007/s00248-008-9469-5 19018587

[B9] Fabiano M. Danovaro R. (1998). Enzymatic activity, bacterial distribution, and organic matter composition in sediments of the ross sea (Antarctica). Appl. Environ. Microbiol. 64, 3838–3845. 9758808 10.1128/aem.64.10.3838-3845.1998 PMC106564

[B10] Herbert R. A. (1999). Nitrogen cycling in coastal marine ecosystems. FEMS Microbiol. Rev. 23, 563–590. 10.1111/j.1574-6976.1999.tb00414.x 10525167

[B11] Kim Y. O. Khosasih V. Nam B. H. Lee S. J. Suwanto A. Kim H. K. (2012). Gene cloning and catalytic characterization of cold-adapted lipase of Photobacterium sp. MA1-3 isolated from blood clam. J. Biosci. Bioeng. 114, 589–595. 10.1016/j.jbiosc.2012.06.013 22841866

[B12] Kimura M. (1980). A simple method for estimating evolutionary rates of base substitutions through comparative studies of nucleotide sequences. J. Mol. Evol. 16, 111–120. 10.1007/BF01731581 7463489

[B13] Li X. Yuan H. Li N. Song J. (2008). Organic carbon source and burial during the past one hundred years in Jiaozhou Bay, North china. J. Environ. Sci. (China). 20, 551–557. 10.1016/S1001-0742(08)62093-8 18575107

[B14] Liu S. M. Zhang J. Chen H. T. Zhang G. S. (2005). Factors influencing nutrient dynamics in the eutrophic Jiaozhou Bay, North China. Progr. Oceanogr. 66, 66–85. 10.1016/j.pocean.2005.03.009

[B15] Liu S. M. Zhu B. D. Zhang J. Wu Y. Liu G. S. Deng B.. (2010). Environmental change in Jiaozhou Bay recorded by nutrient components in sediments. Mar. Pollut. Bull. 60, 1591–1599. 10.1016/j.marpolbul.2010.04.003 20427060

[B16] Liu X. F. Cao Y. Zhang H. L. Chen Y. J. Hu C. J. (2015b). Complete Genome Sequence of *Vibrio alginolyticus* ATCC 17749^T^. Genome Announc. 3:e01500-14. 10.1128/genomeA.01500-14 25635021 PMC4319515

[B17] Liu X. Hu H.-W. Liu Y.-R. Xiao K.-Q. Cheng F.-S. Li J. (2015a). Bacterial composition and spatiotemporal variation in sediments of Jiaozhou Bay, China. J. Soils Sedim. 15, 732–744. 10.1007/s11368-014-1045-7

[B18] Liu Z. Wei H. Liu G. Zhang J. (2004). Simulation of water exchange in Jiaozhou Bay by average residence time approach. Estuar. Coast. Shelf Sci. 61, 25–35. 10.1016/j.ecss.2004.04.009

[B19] Lloyd K. G. Schreiber L. Petersen D. G. Kjeldsen K. U. Lever M. A. Steen A. D.. (2013). Predominant archaea in marine sediments degrade detrital proteins. Nature 496, 215–218. 10.1038/nature12033 23535597

[B20] Mansson M. Nielsen A. Kjærulff L. Gotfredsen C. H. Wietz M. Ingmer H.. (2011). Inhibition of virulence gene expression in Staphylococcus aureus by novel depsipeptides from a marine photobacterium. Mar. Drugs 9, 2537–2552. 10.3390/md9122537 22363239 PMC3280567

[B21] Moore E. K. Harvey H. R. Faux J. F. Goodlett D. R. Nunn B. L. (2014). Electrophoretic extraction and proteomic characterization of proteins buried in marine sediments. Chromatography 1, 176–193. 10.3390/chromatography1040176

[B22] Olivera N. L. Sequeiros C. Nievas M. L. (2007). Diversity and enzyme properties of protease-producing bacteria isolated from sub-Antarctic sediments of Isla de Los Estados, Argentina. Extremophiles 11, 517–526. 10.1007/s00792-007-0064-3 17487446

[B23] Patel A. B. Fukami K. Nishijima T. (2001). Extracellular proteolytic activity in the surface sediment of a eutrophic inlet. Microb. Environ. 16, 25–35. 10.1264/jsme2.2001.25

[B24] Saitou N. Nei M. (1987). The neighbor-joining method: a new method for reconstructing phylogenetic trees. Mol. Biol. Evol. 4, 406–425. 3447015 10.1093/oxfordjournals.molbev.a040454

[B25] Talbot V. Bianchi M. (1997). Bacterial proteolytic activity in sediments of the Subantarctic Indian Ocean sector. Deep Sea Res. II 44, 1069–1084. 10.1016/S0967-0645(96)00107-5

[B26] Tamura K. Peterson D. Peterson N. Stecher G. Nei M. Kumar S. (2011). MEGA5: molecular evolutionary genetics analysis using maximum likelihood, evolutionary distance, and maximum parsimony methods. Mol. Biol. Evol. 28, 2731–2739. 10.1093/molbev/msr121 21546353 PMC3203626

[B27] Thamdrup B. Dalsgaard T. (eds.). (2008). Nitrogen Cycling in Sediments. Hoboken, NJ: John Wiley & Sons, Inc.

[B28] Urbanczyk H. Ast J. C. Dunlap P. V. (2011). Phylogeny, genomics, and symbiosis of Photobacterium. FEMS Microbiol. Rev. 35, 324–342. 10.1111/j.1574-6976.2010.00250.x 20883503

[B29] Wakeham S. G. Lee C. Hedges J. I. Hernes P. J. Peterson M. J. (1997). Molecular indicators of diagenetic status in marine organic matter. Geochim. Cosmochim. Acta 61, 5363–5369. 10.1016/S0016-7037(97)00312-8

[B30] Zhao G. Y. Chen X. L. Zhao H. L. Xie B. B. Zhou B. C. Zhang Y. Z. (2008). Hydrolysis of insoluble collagen by deseasin MCP-01 from deep-sea Pseudoalteromonas sp. SM9913: collagenolytic characters, collagen-binding ability of C-terminal polycystic kidney disease domain, and implication for its novel role in deep-sea sedimentary particulate organic nitrogen degradation. J. Biol. Chem. 283, 36100–36107. 10.1074/jbc.M804438200 18977758 PMC2662289

[B31] Zhao H. L. Chen X. L. Xie B. B. Zhou M. Y. Gao X. Zhang X. Y. (2012). Elastolytic mechanism of a novel M23 metalloprotease pseudoalterin from deep-sea Pseudoalteromonas sp. CF6-2: cleaving not only glycyl bonds in the hydrophobic regions but also peptide bonds in the hydrophilic regions involved in cross-linking. J. Biol. Chem. 287, 39710–39720. 10.1074/jbc.M112.405076 23012370 PMC3501066

[B32] Zhou M. Y. Chen X. L. Zhao H. L. Dang H. Y. Luan X. W. Zhang X. Y.. (2009). Diversity of both the cultivable protease-producing bacteria and their extracellular proteases in the sediments of the South China sea. Microb. Ecol. 58, 582–590. 10.1007/s00248-009-9506-z 19301066

[B33] Zhou M. Y. Wang G. L. Li D. Zhao D. L. Qin Q. L. Chen X. L.. (2013). Diversity of both the cultivable protease-producing bacteria and bacterial extracellular proteases in the coastal sediments of King George Island, Antarctica. PLoS ONE 8:e79668. 10.1371/journal.pone.0079668 24223990 PMC3817139

